# Cancer Risks Associated with Arsenic: Lamm et al. Respond

**DOI:** 10.1289/ehp.9927R

**Published:** 2007-07

**Authors:** Steven H. Lamm, Arnold Engel, Manning Feinleib, Cecelia A. Penn, Rusan Chen

**Affiliations:** Consultants in Epidemiology and Occupational Health LLC, Washington, DC, E-mail: Steve@CEOH.com; Johns Hopkins University Bloomberg, School of Public Health, Baltimore, Maryland; Georgetown University Graduate School, Washington, DC

We thank Guo for his comments and additional information. As indicated by Guo in his letter, three of the six southwest Taiwan townships in in the internal cancer study of Wu et al. (1989) (townships 2, 4, and 6) show significant dose–response relationships for bladder and lung cancer standardized mortality ratios (SMRs) with respect to the median village well arsenic levels, whereas the other three townships (townships 0, 3, and 5) show high background rates for these cancers and no significant dose–response relationship with village arsenic level. Figure 1 demonstrates that the township-specific inverse linear regression lines for townships 2, 4, and 6 all meet the no increased risk level of SMR = 100 (inflection point) at arsenic exposure levels of approximately 125–150 μg/L, which is consistent with a threshold model. That is the same inflection point range seen for skin cancer prevalence in southwest Taiwan (Byrd et al. 1996) and in Inner Mongolia (Lamm et al., in press).

In contrast, the data for townships 0, 3, and 5 are indicative of high background bladder and lung cancer rates (SMRs > 250 at low arsenic levels) that are independent of the arsenic level. We inferred from these analyses the presence of a second (non-arsenic) carcinogenic factor and speculated that it might be related to the nonarsenic etiological factors for blackfoot disease, a condition uniquely reported for this area. On the basis of ongoing analyses, we are currently less inclined to believe that the “township” factor is related to blackfoot disease.

Guo inquires whether exposure heterogeneity within the villages has affected the accuracy of the risk estimates based on the village medians and suggests using alternative exposure indicators. We have examined this. The analytic fits to the models demonstrated in Figure 1 are quite similar whether the median or the mean is used as the summary exposure indicator for the villages; Table 1 shows the robustness of the arsenic concentration of the inflection point with the use of a variety of exposure indicators. The table demonstrates that the inflection point for this group of townships and its 95% confidence interval (CI) for these townships is also robust, based on 20 villages. The lower confidence limit of the inflection point is 40μg/L arsenic.

In spite of the uncertainties in the exposure assessments, the analytic findings are quite robust. They best fit a nonlinear or threshold carcinogenic risk model for arsenic with an inflection point at 150 μg/L (Taiwan) with the presence of at least one additional confounding risk factor. Further analysis will follow the deciphering of the village code. However, interpretation should be cautious because the Wu et al. (1989) study contained data for only about one-third of the villages in the six-township area.

## Figures and Tables

**Figure 1:**
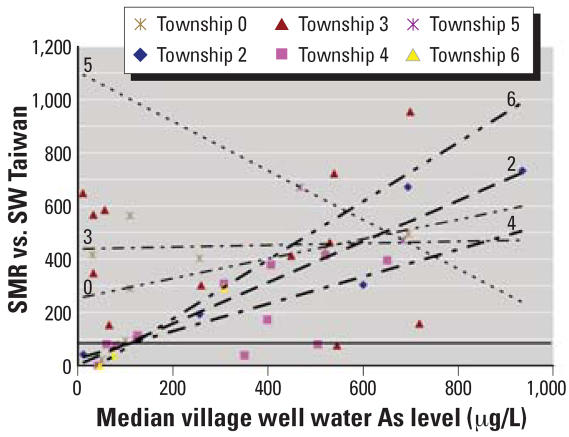
SMRs for bladder and lung cancer by median village well arsenic level for the 42 villages in the six townships in the southwest Taiwan study of Wu et al. (1989) with linear regression analysis by township. SMR = 100 is the level of no increased risk with southwest Taiwan as the reference population. Townships: 0, I-chu; 2, Pu-tai; 3, Hsieh-chia; 4, Yen-shui; 5, Pei-men; 6, Hsia-ying.

**Table 1:** Inflection points (SMR = 100) in the inverse linear regression model.

Township no. (name)	No. of villages	Median	Mean	Midrange	Maximum
2 (Pu Tai)	5	124.5	130.3	124.4	144.2
4 (Yen Shi)	12	141.3	142.2	146.3	183.4
6 (Hsia Ying)	3	131.2	131.2	131.2	131.2
2,4,6 (95% CI)	20	151.0 (42–229)	151.7 (38–231)	151.5 (31–233)	172.1 (39–271)
